# Case report: Late onset type 3 hemiplegic migraine with permanent neurologic sequelae after attacks

**DOI:** 10.3389/fneur.2024.1359994

**Published:** 2024-02-09

**Authors:** Mantas Jokubaitis, Givi Lengvenis, Birutė Burnytė, Eglė Audronytė, Kristina Ryliškienė

**Affiliations:** ^1^Center of Neurology, Faculty of Medicine, Vilnius University, Vilnius, Lithuania; ^2^Department of Radiology, Nuclear Medicine and Medical Physics, Faculty of Medicine, Institute of Biomedical Sciences, Vilnius University, Vilnius, Lithuania; ^3^Faculty of Medicine, Institute of Biomedical Sciences, Vilnius University, Vilnius, Lithuania

**Keywords:** hemiplegic migraine, SCN1A, headache, brain atrophy, permanent deficits

## Abstract

This case study describes a 57-year-old woman with a six-year history of recurrent episodes characterized by visual, sensory, speech disturbances, hemiparesis and severe one-sided headaches accompanied by fever and altered consciousness. Initially misdiagnosed as a stroke, the atypical disease course and MRI findings led to additional genetic testing which revealed a sodium voltage-gated channel gene mutation (T1174S), confirming a diagnosis of sporadic hemiplegic migraine. The migraine prophylaxis showed some improvement in episode frequency and severity. Despite an initial improvement, the patient underwent severe cognitive decline and developed new permanent neurological symptoms during the subsequent 7 years of follow-up.

## 1 Introduction

Hemiplegic migraine (HM) constitutes a rare subtype of migraine with aura, distinguished by fully reversible episodes of motor weakness (1). While it typically adheres to a transient nature, in some rare instances, the aura symptoms persist for an extended period, or even become permanent. There is a scarcity of literature examining cases of prolonged or permanent HM. In this context, we present a patient with late-onset HM who developed severe brain atrophy and permanent neurologic sequelae after severe attacks.

## 2 Case description

A 57-year-old Caucasian female visited an outpatient clinic following increase in frequency and severity of episodes of visual disturbances (flickering in both eyes, duration 3 to 5 min), sensory disturbances (unilateral numbness of fingers, duration 3 to 5 min), disturbance of speech (unable to comprehend speech or produce words) and subsequent severe one-sided headache (with nausea, vomiting, instability, need for rest) together with disturbance of consciousness and fever of 38–39°C. These episodes commenced at the age of 51, exhibiting an average duration of 3 days and recurring once every 2 to 3 months, typically requiring medical intervention. During the visit, the patient reported an increase in episode frequency (three times per month) and severity (disorientation, speech disturbance and headache lasting for a week). There were no discernible triggers identified for these attacks. Due to the presence of fever and alterations in consciousness and behavior during episodes, she underwent multiple admissions to infectious disease or clinical toxicology departments. An MRI was performed – slight cortical and corpus callosum atrophy along with several subcortical white matter lesions in the left hemisphere were noted.

A stroke diagnosis had been established a year ago, characterized by left hemiparesis, dysarthria, and instability. However, there were no residual symptoms upon discharge, and ischemic changes were absent on MRI. The patient was prescribed clopidogrel (75 mg q.d.). Past medical history included controlled arterial hypertension and hyperlipidemia managed with nebivolol (5 mg q.d.) and atorvastatin (20 mg q.d.). There was no history of trauma, surgery, or pregnancy. Menopause commenced at the age of 50. The patient denied prior usage of hormonal contraceptives or tobacco. Migraine with typical aura had been diagnosed in her grandmother, aunt, and cousin. Additionally, the patient’s father suffered a stroke in old age. Clinical examination unveiled left face hypoesthesia, slight ataxia on the finger-to-nose test, and an ataxic gait. The mini-mental status examination (MMSE) yielded a score of 25/30 (points lost: −1 orientation to time, −1 registration, −2 attention and calculation, −1 recall).

The patient underwent further outpatient testing. A wide array of tests had been performed, including toxicology screening, serologic testing for tick-borne encephalitis, Lyme disease, HIV, syphilis, standard metabolic (including lactate), rheumatologic panels and a serum panel for autoimmune encephalitis, but all of laboratory tests yielded negative results. EEG revealed non-specific findings, and only initial atherosclerotic changes were observed on extracranial arterial duplex scan. No significant alterations were detected on MRI compared to prior scans. The diagnosis of suspected hemiplegic migraine (HM) was established, leading to the prescription of prophylactic treatment with valproic acid (500 mg q.d.).

**Figure 1:**
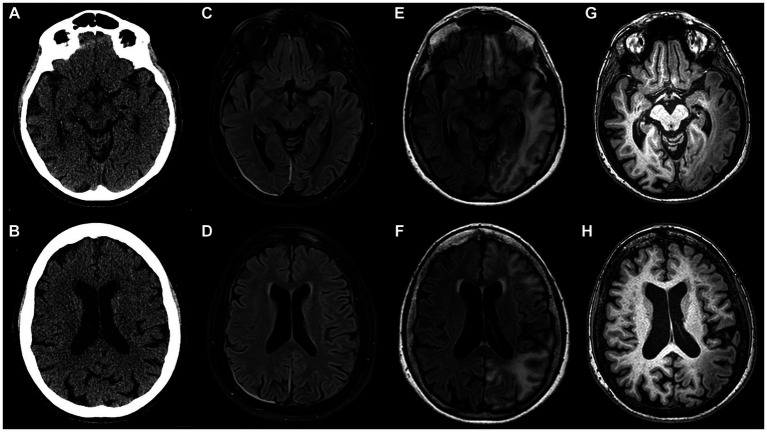
Inconspicuous head CT on admission to the emergency department in 2017 **(A,B)**; brain MRI acquired on day 6 after admission **(C,D)** appears nearly normal (note pachymeningeal thickening and hyperintensity on the right attributed to a recent lumbar puncture); follow-up brain MRI scan performed 12 weeks after initial head CT **(E–H)**: in the left hemisphere extensive juxtacortical and deep white matter high T2 signal lesions on FLAIR **(E,F)**, overlying cortex is atrophic and exhibits higher T1 signal than contralateral normally-appearing grey matter **(G,H)**. Note that lesions are continuous across different vascular territories.

A month later, a subsequent severe episode of prolonged migraine with aura prompted an emergency department referral. Upon admission, multimodal CT with angiography and perfusion were normal (Figures 1A,B, year 2017). The patient was then admitted to the neurology department where her condition continued to deteriorate. She developed a fever of 39°C, right arm paresis, complete motor and partial sensory aphasia along with complete alexia and agraphia. MRI performed on day 6 after the admission was unremarkable (Figures 1C,D). Repeated testing for neuroinfection and autoimmune encephalitis during hospitalization remained negative. A transcranial contrast-enhanced ultrasound revealed no right-to-left shunt. After stabilization, the patient was transferred to rehabilitation. A geneticist consultation revealed a single nucleotide mutation (T1174S) in the sodium voltage-gated channel gene (SCN1A), confirming the diagnosis of type 3 HM.

During a follow-up visit 2 months later, sensorimotor aphasia, right homonymous hemianopia, mild right arm paresis, and ataxic gait persisted. The patient received valproic acid (500 mg b.i.d.) and verapamil (120 mg q.d.) for migraine prevention as well as oral aspirin (1,000 mg q.d.) and intramuscular combination of diclofenac (75 mg), metoclopramide (10 mg), dexamethasone (8 mg) for acute migraine attacks. Follow-up MRI 3 months after the event revealed extensive high T2/dark fluid signal areas in temporo-occipital, frontal, parietal sub-and juxtacortical white matter regions with overlying cortex thinning and elevated T1 signal compared to normal cortex (Figures 1E–H). The changes were not adhering to arterial vascular territories.

**Figure 2:**
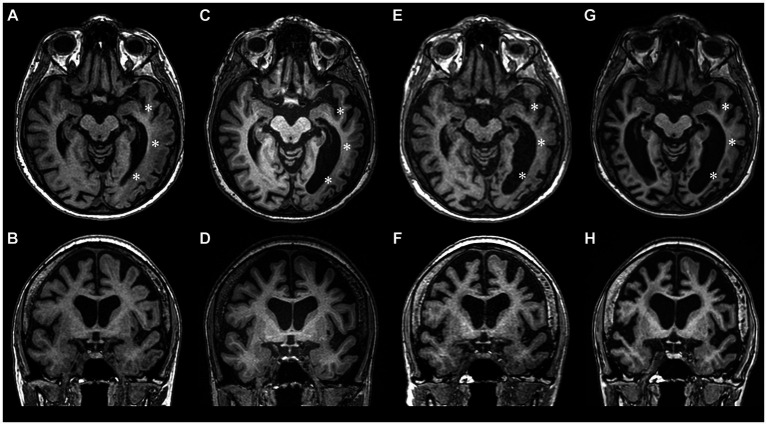
Longitudinal MRI studies during 2017–2023 period showing progression of brain atrophy. **(A,B)** – year 2017, **(C,D)** – year 2018, **(E,F)** – year 2021, **(G,H)** – year 2023. During the follow-up years 2017–2021, atrophy was mostly pronounced in the left temporooccipital region (asterisks), but after the 2022 HM episode, significant atrophy of the right hemisphere also ensued.

Despite improved right arm paresis, subsequent annual follow-ups over 2 years revealed permanent motor-predominant aphasia and progressive cognitive dysfunction (MMSE score 17). The frequency of migraine with aura decreased to one every 3 months and episodes with fever occurred twice a year. During a 4-year MRI follow-up (2017–2021) the progression of brain atrophy continued without any new focal lesions (Figures 2A–F).

**Figure 3:**
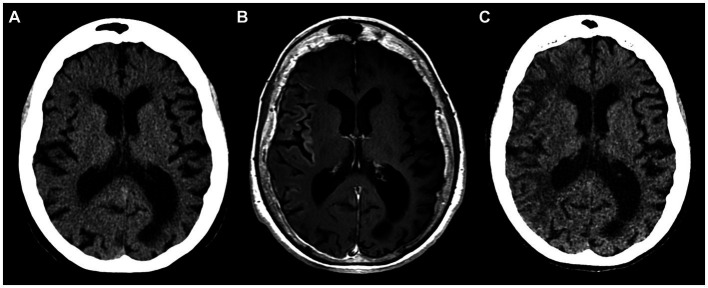
Inconspicuous head CT on admission in 2022 **(A)**; brain MRI with intravenous gadolinium-based contrast agent on day 6 after admission showing subtle cortical enhancement in right insular, frontal, temporal regions **(B)**; follow-up head CT 8 weeks after the initial scan display hypodense white matter lesions in the right hemisphere **(C)**.

The patient did not visit a neurologist for another 3 years, until two consecutive episodes of HM with left arm and leg paresis, fever, and severe cognitive deterioration (MMSE score of 10) occurred in 1 week following a valproic acid dose reduction by the patient (Figures 3A–C, year 2022). EEG revealed non-specific changes without any epileptiform activity.

As of year 2023, the patient remains with severe cognitive impairment (MMSE score of 8), moderate sensorimotor aphasia (patient’s communication is limited to single words, one-step commands, perseverations are noted), severe apraxia, grasp reflex, mild right arm paresis and a slightly ataxic gait. Managed with valproic acid (500 mg b.i.d.), verapamil (120 mg q.d.) and aspirin (1,000 mg q.d.), the frequency of mild HM episodes decreased to two per year. The latest follow-up MRI images are shown in Figures 2G,H.

## 3 Discussion

Our report outlines a case of late-onset type 3 HM, characterized by permanent neurological sequelae, progressive cognitive decline, and substantial brain MRI changes after HM attacks, accompanied by a progressive pattern of brain atrophy. A remarkably analogous case was documented by Hayashi et al. in 1997, detailing the admission of a 44-year-old patient with a history of prolonged HM episodes (2). In their case, during a severe episode, the patient manifested dysphasia, confusion, and uncooperativeness, accompanied by a fever of 39°C. Ictal MRI revealed right cerebral hemiatrophy, while SPECT did not indicate any perfusion disturbances. A month later, MRI exhibited cortical findings akin to those observed in our case. Following the episode, the patient became permanently demented and required constant care. The patient in Hayashi’s case, along with two other cases documented in the literature where HM presented as encephalopathy, fever, and resulted in permanent neurological deficits, were not subjected to genetic testing for alterations in the SCN1A gene (2–4).

While atrophic brain changes have been reported in patients with the CACNA1A mutation, our patient has a mutation in the SCN1A gene, a genetic alteration not previously linked to such severe brain atrophy in HM patients (5–7). Consistent with previous findings (2, 8), imaging changes in our patient emerged later in the disease course, with the most severe MRI alterations appearing after the HM symptoms had subsided. Notably, substantial brain atrophy was observed over the course of the disease, with the most profound changes occurring after two severe attacks.

The underlying reasons for these changes are not completely understood. Loss-of-function mutations of the SCNA1A gene are typically associated with various epilepsy phenotypes due to inhibitory neuronal hypoexcitability, while gain-of-function mutations are linked to HM, leading to neuronal hyperexcitability and prolonged depolarization associated with cortical spreading depression (CSD) (9, 10). Experimental and computational models have provided insight into the T1174S mutation identified in our case, revealing divergent functional effects associated with both a gain and a loss of function of the sodium channel (9). Cestèle et al. explained this phenomenon by the sensitization of the mutant sodium channel to neuromodulation rather than the intrinsic gating property of the channel, suggesting that the effects of the mutation may be influenced by parental genetic factors.

In the context of HM, the long-lasting depolarization of inhibitory neurons leads to the depletion of chloride ions and the accumulation of extracellular potassium (9). This disequilibrium may result in generalized neuronal depolarization, massive release of glutamate, and consequently, the induction of CSD. The notion that prolonged aura and CSD may trigger an elevated metabolic demand, subsequently causing cortical swelling, contrast enhancement, and dysregulation of the blood–brain barrier, has been postulated for quite some time (11). Indeed, the transient cortical enhancement observed on MRI after the 2022 attack (Figure 3), could be attributed to a disrupted blood–brain barrier – a finding uncommon in HM that might also be related to the severity of the attack (12). Furthermore, the irreversible changes seen in our patient could be attributed to cytotoxic edema as an extreme expression of CSD, ultimately leading to cell death (7). Alternatively, histologic examination of the HM case reported by Pellerin et al. revealed signs of non-inflammatory angiopathy, potentially contributing to late-phase atrophic changes (12).

A comprehensive analysis of literature regarding HM linked to persistent or permanent neurological symptoms revealed that around half of the cases exhibited an onset of HM at a significantly later age than the typical manifestation in the first two decades of life (3, 4, 7, 13–15). To our knowledge, no research has been conducted to elucidate the underlying mechanisms of this observation. We hypothesize that the atrophic brain changes might be linked to older age and a more pronounced impact of the neurovascular uncoupling phenomenon (i.e., a mismatch between brain activity and blood flow), rather than being directly attributed to HM type *per se* (16).

No randomized controlled trials have investigated medications for both acute and preventative therapies for HM. Treatment for HM is based on empirical experience with medications used for more common migraine types. Limited evidence from single reports and small cohort studies suggests potential effectiveness of nasal ketamine, intravenous furosemide, magnesium, and verapamil for acute headache and aura treatment (17). Medications with low-quality evidence for HM prevention include oral verapamil, acetazolamide, flunarizine, lamotrigine, and propranolol (17). In our case, HM management with valproic acid (500 mg b.i.d.) and verapamil (120 mg q.d.) resulted in a reduction in episode frequency and severity. Conversely, an arbitrary reduction in valproic acid dosage from 1,000 mg to 500 mg led to two subsequent severe episodes with permanent neurologic sequelae.

Our case report is not without limitations. Firstly, we did not conduct testing for NOTCH3 mutations, limiting our investigation to an HM genetic panel. While the migrainous headache and dementia observed in our case might exhibit similarities with cerebral autosomal dominant arteriopathy with subcortical infarcts and leukoencephalopathy (CADASIL), the diagnosis is unlikely due to the gradual onset of motor symptoms, prolonged consciousness disturbances and the absence of multiple small subcortical infarctions. Secondly, the follow-up MRI after the severe episode in 2017 was conducted after 3 months. Consequently, the exact temporal development of the pronounced left hemisphere changes remains uncertain. This temporal gap introduces ambiguity regarding the precise onset and progression of structural MRI findings. Lastly, genetic testing on family members with a history of migraine was unattainable, as all of them were deceased. In addition, the patient did not have any siblings or children. The inability to perform genetic testing on these individuals hinders our ability to explore potential hereditary factors contributing to the observed clinical manifestations.

In conclusion, our case presented highlights several important aspects to consider when managing HM patients with permanent neurologic and radiologic sequelae. More research is needed to understand the mechanisms underlying permanent changes in HM.

## Data availability statement

The raw data supporting the conclusions of this article will be made available by the authors, without undue reservation.

## Ethics statement

Written informed consent was obtained from the individual(s) for the publication of any potentially identifiable images or data included in this article.

## Author contributions

MJ: Data curation, Writing – original draft, Writing – review & editing. GL: Data curation, Resources, Visualization, Writing – original draft, Writing – review & editing. BB: Writing – original draft, Writing – review & editing. EA: Investigation, Writing – original draft, Writing – review & editing. KR: Methodology, Supervision, Writing – original draft, Writing – review & editing.
